# A Duplicated Anterior Inferior Cerebellar Artery Traversing a Bifurcated Abducens Nerve

**DOI:** 10.7759/cureus.106516

**Published:** 2026-04-06

**Authors:** George Triantafyllou, Panagiotis Papadopoulos-Manolarakis, Maria Piagkou

**Affiliations:** 1 Department of Anatomy, School of Medicine, Faculty of Health Sciences, National and Kapodistrian University of Athens, Athens, GRC

**Keywords:** abducens nerve, anatomical variation, anterior inferior cerebellar artery, cerebellopontine angle, neurovascular conflict

## Abstract

The neurovascular relationship between the anterior inferior cerebellar artery (AICA) and the abducens nerve (CN VI) exhibits significant morphological variability, with important implications for skull base surgery and neurology. During routine cadaveric dissection of a 68-year-old male donor, an uncommon anatomical configuration was identified. The left side demonstrated a duplicated AICA originating from the basilar artery. While the first branch (AICA-1) coursed ventrally to CN VI, the second branch (AICA-2) passed directly through a bifurcation of the nerve. The contralateral side showed a single AICA following a typical ventral course relative to CN VI. Awareness of such variations is essential to reduce the risk of iatrogenic injury during cerebellopontine angle procedures and to facilitate the recognition of potential neurovascular conflict syndromes. Careful preoperative evaluation using high-resolution magnetic resonance imaging may aid in identifying these relationships.

## Introduction

The vascular anatomy of the posterior cranial fossa exhibits significant morphological variability, especially with respect to the branches of the vertebrobasilar system. The intradural segment of the vertebral arteries (VAs) primarily gives rise to the posterior inferior cerebellar arteries (PICAs) before fusing to form the basilar artery (BA). This fusion subsequently gives rise to the anterior inferior cerebellar artery (AICA) proximally and the superior cerebellar artery (SCA) distally [1-3].

The AICA serves as a vital vascular structure within the posterior cranial fossa, providing essential perfusion to the brainstem, cerebellum, and inner ear. Specifically, it typically supplies the inferolateral aspect of the pons and the middle cerebellar peduncle, regions critical for the transmission of motor and sensory information between the cerebrum and cerebellum [4]. The AICA maintains an intimate relationship with the cranial nerves of the cerebellopontine angle. Although its traditional description involves a single trunk passing ventrally or dorsally to the abducens nerve (CN VI), variations such as duplication, triplication, or anomalous branching patterns are frequently encountered in clinical practice [4]. The relationship between the AICA and CN VI is particularly relevant in skull base surgery and neurology [4].

Although AICA [4] and CN VI duplications [5] are commonly documented separately, their concomitance is significantly infrequent. Thus, in this report, we describe such an uncommon neurovascular configuration observed during a standard educational dissection. Its anatomical features and potential clinical implications are discussed below.

## Case presentation

During the skull base dissection of a 68-year-old male cadaver, an unusual neurovascular relationship was identified. The specimen was obtained through the affiliated institution's Body Donation Program [6]. A standard craniotomy was performed, followed by the removal of the cerebral hemispheres at the level of the midbrain. The dura mater of the skull base was then carefully removed to expose the cerebellum and adjacent neurovascular structures. Subsequent dissection was carried out with attention to preserving anatomical relationships. No evidence of trauma or structural abnormality was noted in the head and neck region.

**Figure 1:**
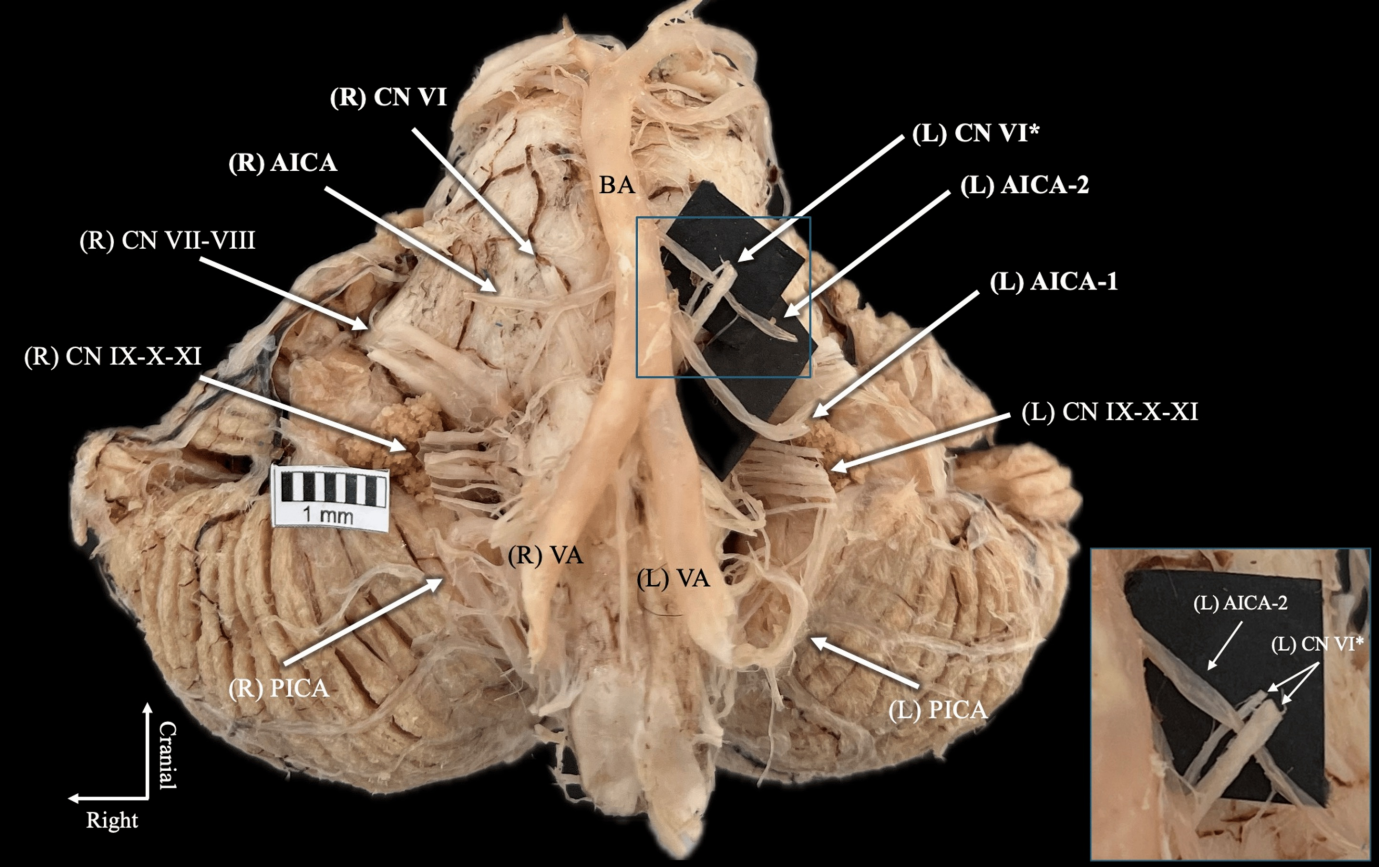
Posterior cranial fossa dissection demonstrating a duplicated left anterior inferior cerebellar artery (AICA) in relation to a bifurcated abducens nerve (CN VI*) One arterial branch (AICA-1) follows a typical ventral course relative to the nerve, whereas the second branch (AICA-2) traverses between the two nerve rootlets. The inset highlights the intraneural passage of the arterial branch. AICA: anterior inferior cerebellar artery; BA: basilar artery; VA: vertebral artery; PICA: posterior inferior cerebellar artery; CN VII-VIII: facial and vestibulocochlear nerves; CN IX-XI: glossopharyngeal, vagus, and accessory nerves

The VAs fused normally to form the BA, and bilateral PICAs originated from their intracranial segments. At a distance of 8.4 mm from its formation, the BA gave rise to the left AICA with a diameter of 1.43 mm. A second AICA branch (1.26 mm diameter) was identified 5.6 mm distal to the first, indicating duplication. The first branch coursed ventral to the abducens nerve, whereas the second branch, located 6.9 mm from its origin, passed through a bifurcation of CN VI (Figure 1). The bifurcation of the nerve was located at the pontomedullary sulcus. This intraneural course is clearly demonstrated in the figure, where the arterial branch traverses between two distinct nerve rootlets.

On the contralateral side, a single AICA arose 7.7 mm from the BA and followed a typical ventral course relative to the abducens nerve. No additional neurovascular variations were observed.

## Discussion

The classic anatomical description of the CN VI involves a single trunk emerging from the pontomedullary sulcus and coursing toward the orbit [5,7]. However, anatomical studies report nerve duplication or splitting in 5-28.6% of cases [5,8]. Nathan et al. [7] categorized these variations into distinct patterns: Pattern 2 involves a single root that splits in the subarachnoid space (6%), while Pattern 3 consists of two separate roots merging in the cavernous sinus (7.5%). Wysiadecki et al. [5] identified a similar duplication in 10% of specimens.

The AICA also exhibits significant morphological diversity. While it typically originates as a single branch from the BA (85.5-90.1%), duplication occurs in approximately 10.4-22.7% of the population [4,9], whereas triplication is even rarer, occurring in 0.7-2.3% of cases [4,9]. These arterial variants often have complex relationships with adjacent cranial nerves in the cerebellopontine angle.

The specific relationship described here, namely, the passage of the AICA through the CN VI, is a documented but uncommon phenomenon. Esmer et al. [9] observed that the AICA pierced the CN VI in three investigated hemispheres, although only a single photographic example was provided. Embryologically, these variations stem from the early developmental stages of the hindbrain. AICA duplication typically results from the failure of fusion or persistence of primitive longitudinal neural arterial channels during the formation of the basilar system [4]. Concurrently, a bifurcated CN VI arises when the multiple initial rootlets emerging from the metencephalon fail to coalesce into a single trunk [5,9], which allows the developing arterial branch to become "trapped" between the neural bundles as the neurovascular architecture is established.

The reported configuration in which a duplicated AICA traverses a bifurcated abducens nerve is uncommon in clinical practice. Canovetti et al. [8] described a similar case in a 62-year-old patient who presented with diplopia and reported an AICA branch passing through an intracisternal duplication of CN VI. Additional clinical reports have demonstrated that vascular compression of the abducens nerve, including involvement of the AICA and VA, may contribute to symptomatic nerve dysfunction [10,11].

From a clinical perspective, these anatomical variants have direct relevance for neuroradiologists, neurologists, and neurosurgeons. Neurovascular conflict involving CN VI, although less frequent than trigeminal nerve compression, may lead to isolated palsy and horizontal diplopia [8,12]. Recent surgical series have shown that microvascular decompression can lead to significant clinical improvement when a clear vascular-neural conflict is identified [12]. In symptomatic cases where conservative management fails, microvascular decompression has been associated with favorable outcomes, including complete remission in a substantial proportion of patients [12]. These findings support the concept that selected cases of abducens nerve palsy may have a reversible vascular etiology.

From a surgical standpoint, such variations are not merely descriptive but may directly influence operative strategy. Unexpected duplication of CN VI increases the risk of iatrogenic injury, particularly if an additional nerve rootlet is not recognized during dissection [5,13]. In addition, intraneural or inter-rootlet arterial courses, as observed in the present case, may increase the technical complexity of surgical exposure and decompression. In microvascular decompression for CN VI palsy, the intraneural course of the artery creates an anatomical complexity that limits traditional vessel mobilization. Attempting to displace the AICA may exert dangerous traction on the nerve rootlets, potentially worsening the deficit [4,9]. Furthermore, the surgeon may inadvertently transect a secondary nerve rootlet if it is mistaken for an arachnoid adhesion during the search for the conflict site [4,9]. Similarly, during the resection of vestibular schwannomas, the tumor's mass effect can further distort this complex anatomy, making the bifurcated nerve and its entrapped arterial branch highly vulnerable. The bifurcated nerve increases the likelihood of iatrogenic injury during tumor debulking, while the lack of vascular mobility makes the AICA branch prone to avulsion or stretching [5]. Given that these duplicated branches may provide critical supply to the brainstem or cerebellum, any injury to this specific neurovascular complex significantly elevates the risk of postoperative ischemic events [5].

Preoperative assessment of these relationships using high-resolution balanced steady-state free precession magnetic resonance imaging (MRI) sequences (such as FIESTA (GE HealthCare, Chicago, Illinois, United States), CISS (Siemens Healthineers, Erlangen, Germany), or TrueFISP (Siemens Healthineers, Erlangen, Germany), depending on the vendor) may enable more precise anatomical delineation of cranial nerves and adjacent vascular structures and improve surgical safety. However, small-caliber arterial branches passing between closely apposed nerve rootlets may be difficult to visualize preoperatively.

Although this case report offers a detailed anatomical investigation, a few limitations should be mentioned. Firstly, as a single-case study involving a cadaveric specimen, these findings represent an isolated observation and cannot be used to establish the prevalence of this variation within the general population. Secondly, the lack of a detailed premortem clinical history for the donor precludes a direct correlation between the observed anatomy and potential symptomatic manifestations.

## Conclusions

The identification of a duplicated AICA traversing CN VI represents an uncommon neurovascular configuration that may be associated with clinical manifestations. Although this anatomical variant is infrequently encountered, clinicians should remain aware of its potential role in neurovascular conflict and should carefully evaluate these relationships. Therefore, comprehensive MRI assessment with multiplanar reconstruction and high-resolution sequences should be prioritized in atypical clinical cases.
